# Organic bases catalyze the synthesis of urea from ammonium salts derived from recovered environmental ammonia

**DOI:** 10.1038/s41598-020-59795-6

**Published:** 2020-02-18

**Authors:** Yuichi Manaka, Yuki Nagatsuka, Ken Motokura

**Affiliations:** 1 School of Material and Chemical Technology, Tokyo Institute of Technology, 4259 Nagatsuta-cho, Midori-ku, Yokohama, Kanagawa 226-8502 Japan; 2 Renewable Energy Research Center, National Institute of Advanced Industrial Science and Technology, 2-2-9 Machiikedai, Koriyama, Fukushima 963-0298 Japan

**Keywords:** Sustainability, Carbon capture and storage

## Abstract

Ammonia from sewage and livestock manure is a major environmental pollutant. To consume environmental ammonia, we investigated the organic base-catalyzed synthesis of urea. 1,8-Diazabicyclo[5.4.0]undec-7-ene (DBU) catalyzes the conversion of ammonium carbamate to urea in 35% yield at 100 °C. Moreover, DBU also converts other ammonium salts into urea. A mechanism that involves nucleophilic attack of ammonia following ion exchange is proposed.

## Introduction

**Figure 1:**
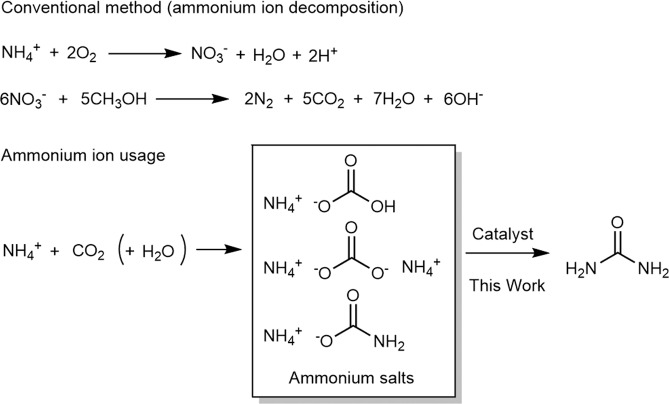
Environmental ammonia treatment methods.

The use of untapped environmental resources with the aim of establishing a sustainable society is attracting increasing attention. Among these resources, sewage and livestock manure have been estimated to contain large amounts of ammonium ions (environmental ammonia)^1–9^. The recovery and use of environmental ammonia represents a possible alternative route to the Haber process for the supply of ammonia. Since environmental ammonia is an environmental pollutant, it is generally decomposed into molecular nitrogen and carbon dioxide at treatment plants with adding of methanol through nitrification and denitrification processes^10^ that involve microorganisms and significant heat energy (Fig. 1). This treatment process also produces N_2_O as a byproduct, which is a well-known greenhouse gas^11–14^. In other words, the treatment of environmental ammonia promotes global warming through the use of additional energy and the production of CO_2_ and N_2_O. Other methods for separating ammonia from wastewater involve ammonia stripping^15–17^ or the use of an ammonia sorbent;^18–21^ while these processes are purposed to remove ammonia, they also require the use of additional bases and heat for operation. In addition, even when ammonia is recovered by ammonia stripping, its purity is low and further purification is required.

If it were possible to recover and reuse environmental ammonia, this unnecessary environmental pollutant becomes a useful chemical resource, which presents a new possible solution for current environmental problems and the supply of an important resource.

Recovering ammonium carbonate, ammonium bicarbonate, or ammonium carbamate by reacting ammonium ions with carbon dioxide (and water) is possibly the least expensive method for recovering environmental ammonia from the perspectives of cost and ammonia condensation. These recovered ammonium salts are not very thermodynamically stable and/or are easily thermally decomposed to ammonia (for reuse as ammonia gas) and carbon dioxide^22^. If the recovered ammonium salts can be directly converted into urea with less additional energy input, which is a more useful industrial chemical product, the usefulness of this ammonia-recovery process is further increased (Fig. 1).

Urea is a well-known chemical product and is a raw material for fertilizer and polymers^23–25^. Urea has also attracted attention as a solid-state energy carrier in recent years^26,27^. Urea is directly synthesized from carbon dioxide and ammonia (from Haber process) commonly^28–30^. The reaction generally operates at 150 °C and at a pressure of 2 MPa, and the urea-synthesis plant can be sustained because of its vicinity to the ammonia-synthesis plant from which it derives much of its input energy^31^. Due to these energy factors, conventional energy-consuming urea synthesis method is not suitable for environmental ammonia usage. On the other hands, since nitrogen in recovered ammonium ion does not have extreme strong triple bond as N_2_, there is no need additional energy for dividing N_2_ bond like Haber process and is an advantage compared with conventional urea synthesis method via Haber process. It is crucial for urea synthesis reaction to decrease the input energy to be accepted by society because one of the purposes of environmental ammonia usage is to reduce the ammonia treatment energy.

Urea has also been synthesized from ammonium carbamate as the substrate and a Cu catalyst;^32^ in this report, a 54% yield of urea was achieved at 140 °C, 14 MPa, and 3 d of incubation. However, large amounts of energy are still required for the reaction. In other report, urea has been synthesized from ammonium carbamate and ammonium bicarbonate mixtures as the substrate;^33^ in this report, a 48.9% yield of urea was achieved at 165 °C, 3.6 MPa, and 90 min of incubation. Large amounts of energy for heating and compressing are still required for the reaction. For further input energy decrease, efficient catalyst for ammonium salt conversion into urea is required.

Herein, we report the synthesis of urea from ammonium salts under milder conditions than other methods previous reported. The reaction was achieved by using organic base as a catalyst. This study is the first to investigate ammonium salts conversion to urea in order to consume environmental ammonia using organic base catalyst.

## Results

### Catalyst screening

**Figure 2:**
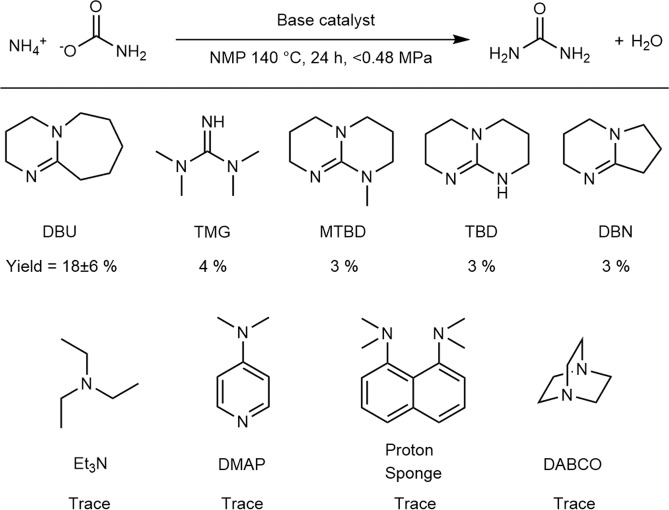
Results of urea synthesis from ammonium carbamate catalyzed by organic base catalysts. Details of the abbreviations used are provided in the experimental section. Experimental conditions: Base (0.38 mmol), ammonium carbamate (3.8 mmol), 140 °C, 24 h, in 1 mL of NMP. Pressure of the inside of the vessel was increased up to 0.48 MPa because of autogenous pressure of thermal decomposition of ammonium carbamate. The amount of produced area was determined by the Fearon reaction (see experimental section).

The first urea-synthesis screening experiments were performed with a variety of organic bases as catalysts and ammonium carbamate as a substrate. The urea-synthesis results using these bases in NMP as the solvent are summarized in Fig. 2. It should be noted that ammonium carbamate decomposes to ammonia and CO_2_ at 59 °C; consequently, some of the ammonium carbamate is converted into these gases in the closed reaction vessel, and the gases increase the inside pressure of the reaction vessel. From Fig. 2, strong bases were observed to catalyze the conversion of ammonium carbamate into urea. No other urea derivative, such as biuret, triuret, or cyanuric acid was detected.

Although a different solvent was used in these experiments, the p*K*_a_ values of the corresponding conjugated acids in AcCN^34,35^ provide valuable insight; bases with conjugate acids with p*K*_a_ values greater than 20 catalyze the conversion of ammonium carbamate into urea (Entries 1–5, Fig. S1). Among them, 1,8-diazabicyclo[5.4.0]undec-7-ene (DBU) showed especially high activity, providing urea in 18% yield (Entry 1, Fig. S1). Although the reason for the high activity of DBU is not clear, it is possible that the stability of DBU in the catalytic reaction plays an important role because TMG and DBN decompose in the reaction solution.

### Solvent effect on urea synthesis

**Figure 3:**
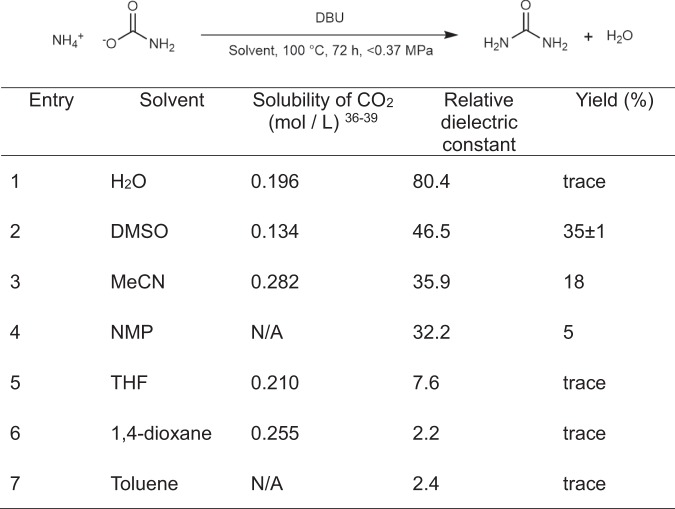
Urea synthesis from ammonium carbamate in various solvents. Experimental conditions: DBU (0.80 mmol), ammonium carbamate (4.0 mmol), 100 °C, < 0.37 MPa, 72 h, in 1 mL of solvent.

We next examined the effect of the solvent in the synthesis of urea using the most active DBU catalyst. Figure 3 lists the urea-synthesis results using solvents with CO_2_ solubility and relative dielectric constant^36–39^. No correlation was observed between CO_2_ solubility and the yield of urea. On the other hand, the relative dielectric constant of the solvent was observed to correlate with the urea yield. Following these two results, this trend appears to be attributable to the solubility of molecular ammonia in the solvent. The maximum yield of 35% was achieved at 100 °C in DMSO as the solvent over a prolonged reaction time (Entry 2, Fig. 3). The solvent without base did not show the efficient catalytic activity (Fig. S2).

### Substrate scope

**Figure 4:**
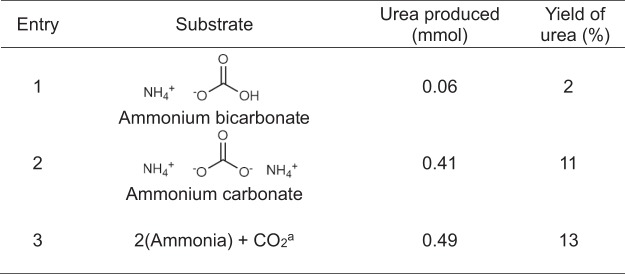
Urea synthesis from possible forms of recovered ammonia. Experimental conditions: DBU (0.36 mol), Ammonium salts (3.6 mmol), 140 °C, 30 h, in 1 mL of DMSO. ^a^Substrate (theoretical value): Ammonia gas (0.72 mmol), CO_2_ (0.36 mmol). The method used to generate ammonia gas and CO_2_ gas is described in the ESI, and the reaction schemes are shown in Fig. S3.

Ammonium carbonate, ammonium bicarbonate, and ammonia gas, which are possible forms of recovered ammonia from the environment, were also employed as substrates for the synthesis of urea in addition to ammonium carbamate, the results of which are shown in Fig. 4. DBU successfully catalyzed the synthesis of urea from the ammonium carbonate despite ammonium carbonate as the substrate generate two water molecules during the reaction (Entries 1, Figs. 4 and S3). Since the generated amount of water is larger than that of the carbamate used as the substrate in the reaction, the equilibrium conversion is lower than that obtained using ammonium carbamate; hence, the lower yield in Entry 1 is attributable to the lower equilibrium conversion. Ammonium bicarbonate also generates more water than ammonium carbamate in this process. Nevertheless, DBU also successfully catalyzed the synthesis of urea from the ammonium bicarbonate. Urea can also be synthesized from gaseous substrates. Ammonia and CO_2_ are the thermal decomposition products of ammonium carbamate, as mentioned above. This result indicates that the decomposition of ammonium carbamate, as the substrate, is likely to play a role through dissolution in the reaction solvent.

### Reaction intermediate measurement

**Figure 5:**
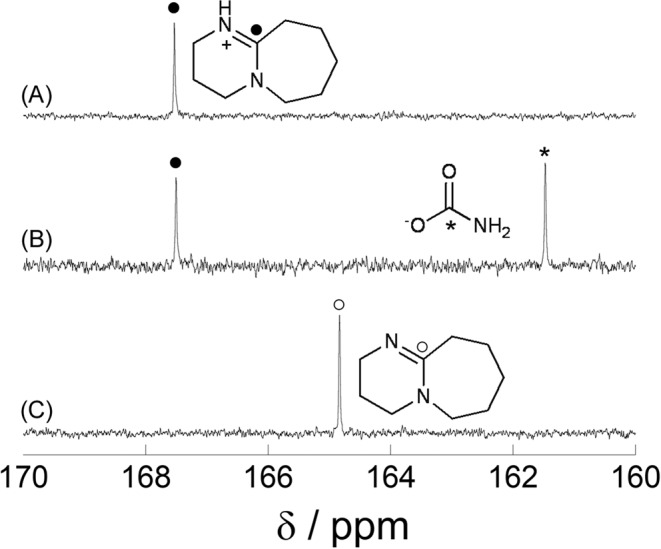
^13^C NMR spectra in methanol-d_4_ of (**A**) DBU + H_2_SO_4_, (**B**) DBU + ammonium carbamate, and (**C**) DBU. Filled circle: quaternary carbon atom of DBUH^+^, Open circle: quaternary carbon atom of DBU, Asterisk: carbonyl carbon atom of carbamate anion.

We used ^13^C NMR and FT-IR spectroscopies to investigate the reaction mechanism involved in the synthesis of urea catalyzed by DBU; the ^13^C NMR spectra are displayed in Fig. 5. As shown in Fig. 5C, the signal of the quaternary carbon in DBU is observed at around 165 ppm. This signal was observed to shift downfield to 167 ppm when ammonium carbamate and DBU were mixed (Fig. 5B). On the other hand, the spectrum of DBU mixed with sulfuric acid (Fig. 5A) showed the same chemical shift as the DBU + ammonium carbamate mixture. Since DBU protonated by sulfuric acid (Fig. 5A) showed the same chemical shift as the DBU with ammonium carbamate, we conclude that the signal observed by mixing DBU with ammonium carbamate is due to protonated DBU, which is also consistent with the shift behavior of protonated DBU in a literature report^40^. On the basis of these results, cation exchange seems to have proceeds to form the DBU salt^41^ of carbamic acid and ammonia. When Et_3_N, which does not produce urea from ammonium carbamate, was employed as the base, this shift phenomenon was not observed (Fig. S4). We have identified that the cation-exchange reaction is important for the efficient synthesis of urea.

**Figure 6:**
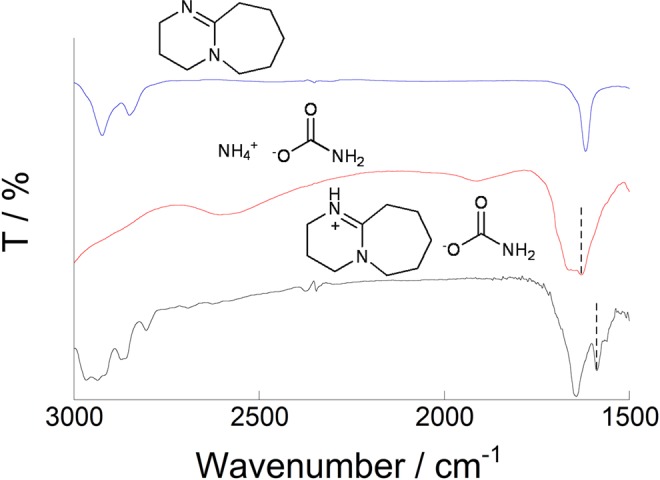
FT-IR spectra of (**A**) DBU, (**B**) ammonium carbamate, and (**C**) DBU + ammonium carbamate. Dashed line: C = O stretch of carbamate anion.

The FT-IR spectra of DBU, ammonium carbamate, and a mixture of DBU and ammonium carbamate are shown in Fig. 6A–C, respectively. A peak assignable to the C = O stretch is observed at around 1628 cm^-1^ in the spectrum of ammonium carbamate. On the other hand, when ammonium carbamate and DBU were mixed, this C = O stretch was observed to shift from 1628 cm^-1^ to 1589 cm^-1^, which is a 40 cm^-1^ shift to lower wavenumber; no such shift was observed when Et_3_N was used (Fig. S5). We conclude that a base that is unable to participate in cation exchange is unable to activate the C = O group^42^.

**Figure 7:**
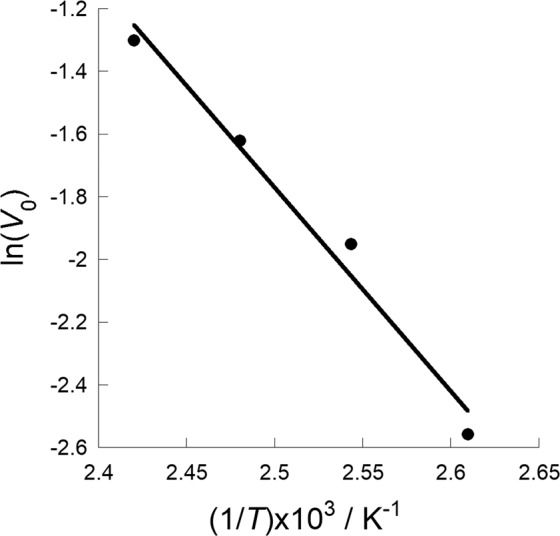
Arrhenius plot for urea synthesis from ammonium carbamate catalyzed by DBU.

Figure 7 shows the Arrhenius plot of the logarithm of the initial urea synthesis rate constant as a function of reciprocal reaction temperature. The initial urea synthesis rate was determined by time-dependence experiments (Figs. S6 and S7) and was observed to increase with increasing reaction temperature. The apparent activation energy for the synthesis of urea using DBU as the catalyst was found to be 54.1 kJ mol^-1^. This value is lower than that of urea synthesis from NH_3_ and CO_2_ under high pressure (94.5 kJ mol^-1^)^43^. Recovered environmental ammonia (ammonia carbamate) and DBU showed the possibility for low energy input urea synthesis.

## Discussion

**Figure 8:**
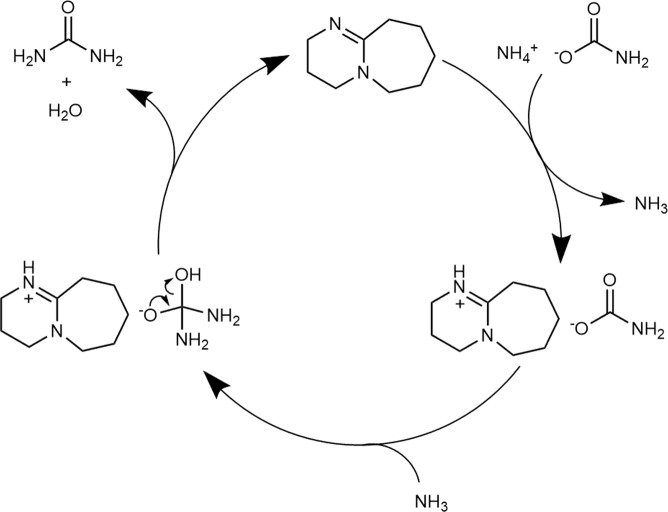
Proposed mechanisms for the DBU catalyzed urea-synthesis reaction from ammonium carbamate.

Based on the NMR and FT-IR results, we propose that DBU is protonated by ammonium carbamate through counter-cation exchange, which weakens the carbamate C = O bond and increases its electrophilicity. The proposed reaction mechanism based on our results is shown in Fig. 8.

At first, DBU undergoes cation exchange with ammonium carbamate to form the DBU salt of carbamate and ammonia. The formed ammonia then nucleophilically attacks the weakened carbonyl group of the carbamate anion, after which urea is formed by dehydration and DBU is regenerated.

Results of solvent effect experiment (Fig. 3) support the proposed reaction mechanism. In the proposed reaction mechanism, molecular ammonia is required to nucleophilically attack the carbonyl group. Therefore, the solubility of ammonia in the solvent may play a key role since the organic base catalyst is dissolved in the reaction solvent.

In summary, we successfully synthesized urea from ammonium carbamate using DBU as an organic base catalyst. In particular, the best yield of 35% was achieved in DMSO as the solvent at 100 °C. The reaction was applicable to other ammonium salts, such as ammonium carbonate and ammonium bicarbonate. NMR and FT-IR spectroscopies reveal that DBU undergoes cation exchange with ammonium carbamate; the activated C = O group of the exchanged salt then undergoes nucleophilic attack by ammonia. Despite the reaction rate with DBU being low, this new catalytic system has the potential to change how environmental ammonia is treated for a sustainable society.

## Methods

### General

Proton nuclear magnetic resonance (^1^H NMR) spectra were recorded on an AVANCE III HD 400 MHz NMR spectrometer (Bruker) or an AVANCE III HD 500 MHz NMR spectrometer (Bruker). Carbon-13 nuclear magnetic resonance (^13^C NMR) spectra were recorded on the AVANCE III HD 400 MHz NMR spectrometer with a Cryoprobe (Bruker). Mass spectra were recorded on a solariX Fourier transform ion cyclotron resonance mass spectrometer (Bruker) or a ACQUITY UPLC system (Waters) or a GCMS-QP2010 SE gas chromatograph mass spectrometer (Shimadzu). Fourier transform infrared (FT-IR) spectra were recorded on a IRTracer-100 Fourier transform infrared spectrophotometer (Shimadzu). UV/Vis spectra were acquired on a UV-2600 UV-Vis spectrophotometer (Shimadzu). The pressure in the pressure tight vessel was measured using a Krone digital pressure gauge KDM30 (Krone).

### Chemicals

1,8-Diazabicyclo[5.4.0]undec-7-ene (DBU), 1,5-diazabicyclo[4.3.0]non-5-ene (DBN), 1,5,7-triazabicyclo[4.4.0]dec-5-ene (TBD), 7-methyl-1,5,7-triazabicyclo[4.4.0]dec-5-ene (MTBD), 1,1,3,3-tetramethylguanidine (TMG), triethylamine (Et_3_N) and ammonium carbamate, were purchased from Tokyo Chemical Industry Co., Ltd. 4-dimethylaminopyridine (DMAP), 1,4-diazabicyclo[2.2.2]octane (DABCO), ammonium carbonate, ammonium bicarbonate, urea were purchased from FUJIFILM Wako Pure Chemical Corporation. 1,8-bis(dimethylamino)naphthalene (proton sponge) was purchased from Sigma-Aldrich Japan. All solvents used were of anhydrous grade, and all chemicals were used as received without further purification.

### Urea synthesis reaction

In a typical reaction, ammonium carbamate (3.8 mmol, 1 eq.) and base (0.38 mmol, 0.1 eq.) were placed in a 20-mL stainless-steel autoclave (TAIATSU Techno) with 1 mL of solvent. The autoclave was set in an oil bath at the desired temperature and the contents were stirred. The pressure inside the autoclave increased to 0.5 MPa during heating due to the NH_3_ and CO_2_ generated by the thermal decomposition of ammonium carbamate inside the vessel. At the required time, the autoclave was immediately quenched by immersion in an ice bath.

When ammonia gas and CO_2_ were used as substrates, an inner cylinder with an open top was used. The base (0.38 mmol, 0.1 eq.) was placed in the inner cylinder with 1 mL of solvent, and ammonium carbamate (3.8 mmol, 1 eq.) was placed in the space between the outside of the inner cylinder and the inside of the outer cylinder. The ammonium carbamate located exterior to the catalyst decomposed to generate gaseous ammonia and CO_2_.

### Urea detection

The urea-synthesis reaction solution was analyzed by GC-MS to qualitatively determine the amount of urea produced. In addition, the concentration of urea was quantitatively determined by the Fearon reaction^44^. For the Fearon reaction, 28.4% aqueous phosphoric acid and 0.4 M aqueous diacetyl monoxime were prepared in deionized water. A 690-μL aliquot of the phosphoric acid solution, 300 μL of the diacetyl monoxime solution, and 10 μL of the sample were placed in a 1.5 mL microtube with a lid-lock. The tube was shaken well for 10 s and then heat at 90 °C for 60 min with slow shaking. As the required time, the tube was immediately quenched by immersion in liquid nitrogen. The reacted solution was subjected to UV/Vis spectroscopy in the 200–800 nm range, and the urea concentration was determined using the calibration curve technique.

## Supplementary information

Supplementary Information
