# Supplementary material for: Organic bases catalyze the synthesis of urea from ammonium salts derived from recovered environmental ammonia
Source: Sci Rep. 2020 Feb 18;10:2834. doi: 10.1038/s41598-020-59795-6 (PMC7028734; doi:10.1038/s41598-020-59795-6)
Supplement: Supplementary file 1 — Supplementary Information [file 41598_2020_59795_MOESM1_ESM.pdf]

Electronic Supplementary Information

for

Organic bases catalyze the synthesis of urea from ammonium salts derived from recovered environmental ammonia

Yuichi Manaka[1,2,*], Yuki Nagatsuka[1], and Ken Motokura[1]

1. *School of Material and Chemical Technology, Tokyo Institute of Technology*

4259 Nagatsuta-cho, Midori-ku, Yokohama, Kanagawa, 226-8502 JAPAN

2. *Renewable Energy Research Center, National Institute of Advanced Industrial Science and Technology*

2-2-9 Machiikedai, Koriyama, Fukushima, 963-0298 JAPAN

*manaka.y.aa@m.titech.ac.jp

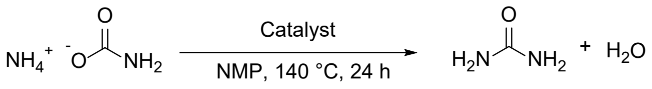

Entry	Base	pK_a values of conjugated acid	Yield (%)
1	DBU	24.3	18
2	TMG	23.4	4
3	TBD	25.5	3
4	MTBD	26.0	3
5	DBN	23.4	3
6	Et_3N	18.8	Trace
7	DMAP	18.0	Trace
8	Proton sponge	N/A	Trace
9	DABCO	N/A	Trace
10	-	-	Trace

Figure S1. Urea synthesis from ammonium carbamate catalyzed by organic bases. Experimental conditions: Base (0.38 mmol), ammonium carbamate (3.8 mmol), 140 °C, 24 h, in 1 mL of NMP. Pressure of the inside of the vessel was increased up to 0.48 MPa because of autogenous pressure of thermal decomposition of ammonium carbamate. The amount of produced area was determined by the Fearon reaction.

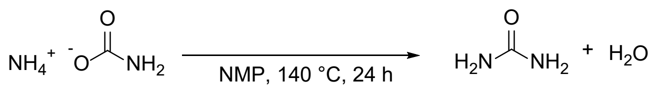

Entry	Solvent	Relative dielectric constant	Yield (%)
1	-	-	0
2	DMSO	46.5	1
3	MeCN	35.9	0
4	NMP	32.2	0
5	THF	7.6	0
6	Toluene	2.4	0
10	1,4-dioxane	2.2	0

Figure S2. Urea synthesis from ammonium carbamate without base.

Experimental conditions: Ammonium carbamate (3.6 mmol), 140 °C, 5 atm, 24 h, in 1 mL of solvent.

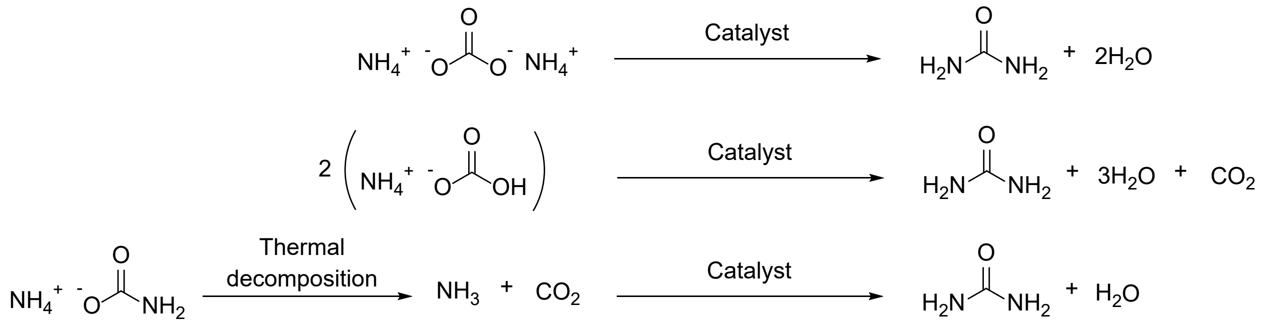

Figure S3. Substrate Scope.

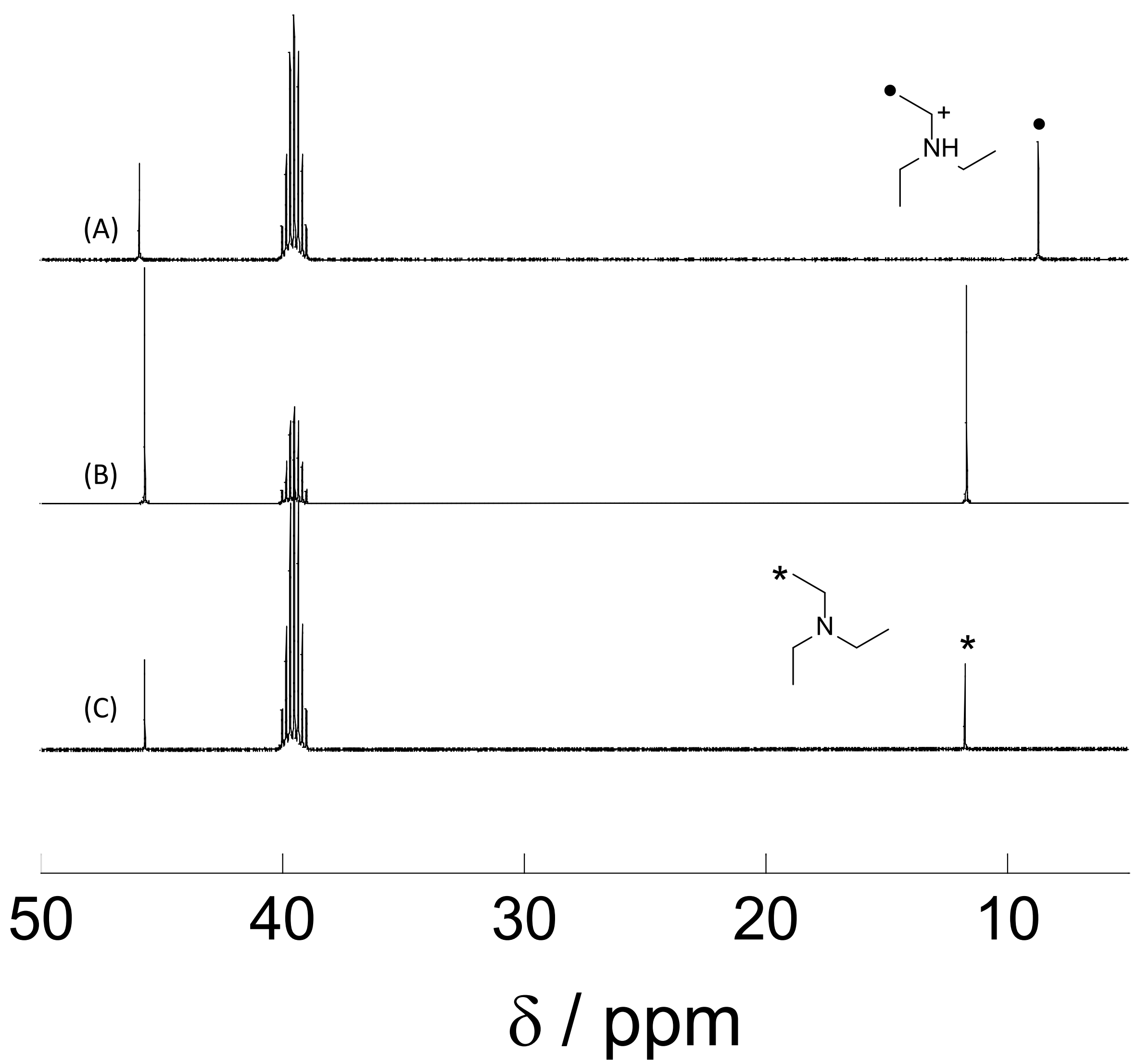

Figure S4. ^{13}C NMR spectra in DMSO of (A) Et_3N + H_2SO_4, (B) Et_3N + ammonium carbamate, and (C) Et_3N. Filled circle: primary atom of Et_3NH^+, Asterisk: primary carbon atom of Et_3N. The signal of the carbon of protonated Et_3N is observed at around 8.7 ppm.

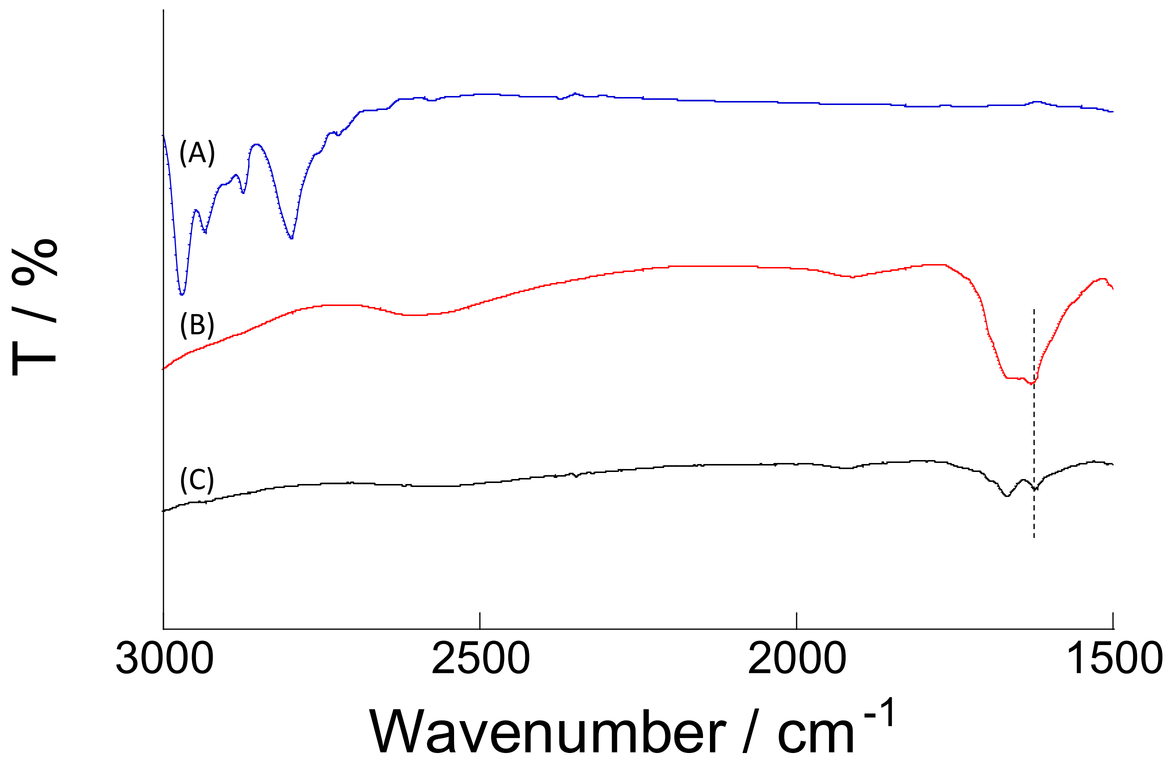

Figure S5. FT-IR spectra of (A) Et_3N, (B) ammonium carbamate, and (C) Et_3N + ammonium carbamate. Dashed line: C=O stretch of carbamate anion.

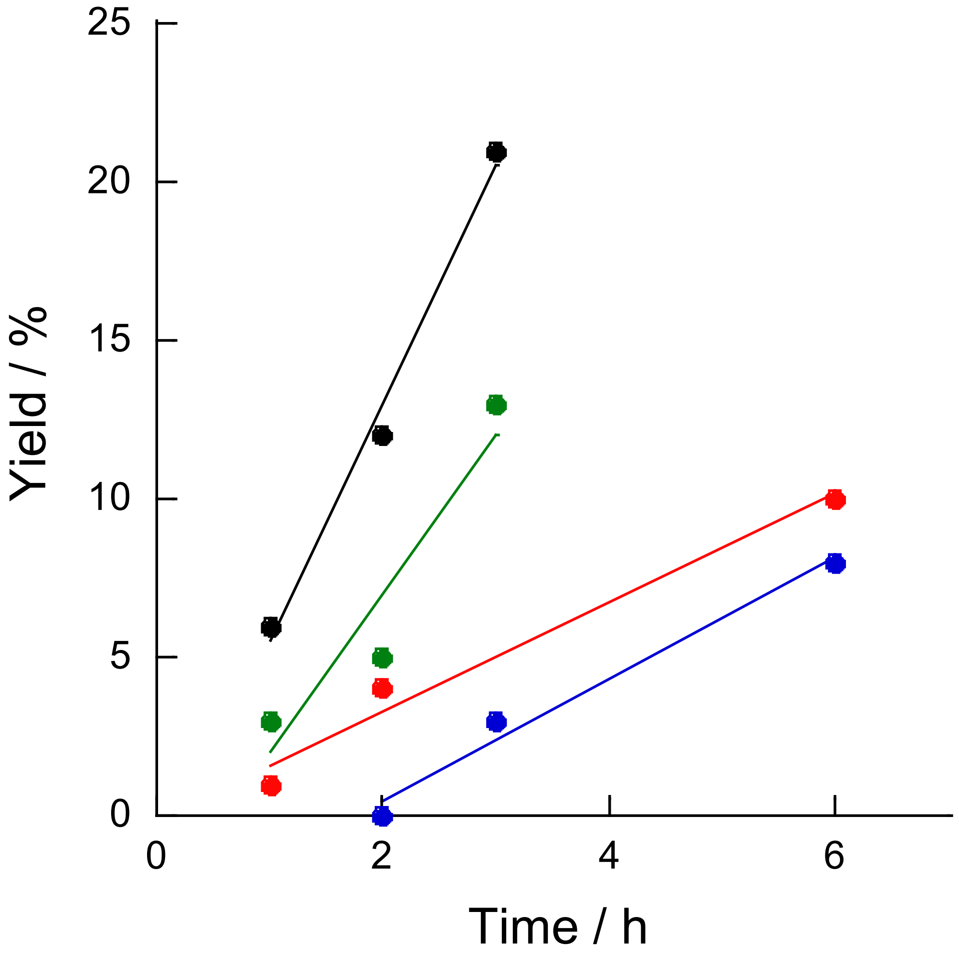

Figure S6. Urea synthesis results under various temperature as a function of time. Experimental conditions: DBU (0.36 mol), ammonium carbamate (3.6 mol), 5 atm, in 1 mL of DMSO. Black (140 °C), Green (130 °C), Red (120 °C), Blue (110 °C).

Entry	Reaction temperature	Slope (%/h)	v_0 (mol/(L*h))	ln(v_0)
1	110 °C	1.96	0.775	-2.56
2	120 °C	3.70	0.142	-1.95
3	130 °C	5.30	0.198	-1.62
4	140 °C	7.47	0.272	-1.30

Figure S7. Urea synthesis results under various temperature.
